# Beyond Screen Time: A Measurement Framework for Behavioral Exposures in Childhood Myopia

**DOI:** 10.3390/life16071178

**Published:** 2026-07-16

**Authors:** Jeong Jun Park, Gwi Eun Yeo, Youra Kim, Joonhyung Kim

**Affiliations:** 1 Department of Anesthesiology and Pain Medicine, CHA Bundang Medical Center, CHA University School of Medicine, Seongnam 13496, Republic of Korea; jeongjun.park@cha.ac.kr; 2 Department of Anesthesiology and Pain Medicine, Bundang Jesaeng General Hospital, Daejin Medical Center, Seongnam 13590, Republic of Korea; sleeplater@naver.com; 3 Green Forest Eye Clinic, Busan 48111, Republic of Korea; 4 Department of Ophthalmology, CHA Bundang Medical Center, CHA University School of Medicine, Seongnam 13496, Republic of Korea

**Keywords:** childhood myopia, outdoor light exposure, near work, digital devices, sleep timing, circadian rhythm, axial length, time-structured exposure

## Abstract

Childhood myopia is increasingly discussed in relation to digital device use, yet total screen duration is biologically nonspecific (not tied to a single biological cause) and may combine outdoor-light deficit, near work intensity, educational routines, and sleep/circadian timing. This critical narrative review used structured database searches to support transparent source identification, not systematic completeness, quantitative pooling, or formal risk-of-bias grading. We narratively appraised evidence by design, exposure specificity, outcome specificity, temporality, and clinical directness, then derived a measurement framework. Outdoor light exposure has the strongest support for preventing incident myopia. Near work and the visual demands of schooling are important, but they are measured in inconsistent ways. Sleep and circadian timing remain biologically plausible, albeit lower-certainty modifiers. Digital device metrics are best interpreted as contextual measurement markers that can recover timing, bout structure, task context, substitution, and adherence, rather than as standalone causal surrogates (stand-ins for a true cause). The proposed framework prioritizes direct measurement of outdoor light, near work intensity, sleep/circadian timing, and axial-growth outcomes, using device-related behavior only when it clarifies daily exposure patterns. This is a narrative review of previously described behavioral factors; it does not identify new risk factors or propose a novel causal model. For measurement, it groups these behaviors by how they occur together across a single day and separates substitution, mediation, interaction, and temporal clustering as distinct estimands (the specific quantities to be estimated). Its purpose is to improve exposure measurement and hypothesis testing, not to establish digital device use, sleep timing, or clustered routines as independent causal risk factors.

## 1. Introduction

### 1.1. Myopia Burden, High-Myopia Complications, and the Digitalization of Childhood and Adolescence

Myopia has become one of the most consequential chronic ocular conditions of childhood and adolescence. The most recent global synthesis indicates that myopia already affects approximately one-third of children and adolescents, with projected case counts exceeding 740 million by 2050 [1]. Global modeling across all age groups further suggests that nearly half of the world population may be myopic and almost one tenth may have high myopia by 2050 [2].

Excessive axial elongation (the eyeball growing too long from front to back) makes this trend clinically important beyond refractive correction alone. Eyes with high myopia are at substantially greater risk of pathologic structural changes (sequelae), including myopic maculopathy, posterior staphyloma, retinal detachment, and other causes of irreversible visual loss. The economic consequences are also considerable, with major productivity loss already attributable to uncorrected myopia and myopic macular disease [3,4].

This burden unfolds in parallel with the rapid digitalization of childhood and adolescence. Educational work, leisure, communication, and entertainment are increasingly delivered through smartphones, tablets, computers, and other digital platforms. These platforms concentrate visual activity indoors, at short viewing distances, and often late in the day. The International Myopia Institute has therefore emphasized that modern myopia risk should be interpreted within a broader environmental framework rather than through isolated behavioral summaries alone [5,6].

### 1.2. Why Screen Time Is an Insufficient Construct

Total screen time is easy to collect but biologically imprecise. It collapses viewing distance, accommodative continuity, ambient illuminance, task context, and time of day into a single duration metric. Children with identical screen-duration totals may therefore have materially different ocular-growth exposures [7,8,9,10].

Outdoor light provides the clearest example of why duration-only device metrics are insufficient. Increased outdoor exposure reduces incident myopia in school-based and randomized studies, and pooled evidence supports a protective association for onset and myopic shift [11,12,13,14,15]. Device-related time may therefore operate partly as a marker of displaced daylight exposure within a fixed 24 h day rather than as a direct optical exposure.

Near work and education studies point to a complementary problem. Total device time is a weak proxy for short viewing distance, long uninterrupted near work, educational load, posture, and timing [7,8,9,16]. Sleep and circadian studies add another layer. Evening screen-based near work may happen in a biologically different context, but the pediatric myopia evidence is still of lower certainty and is confounded by education, indoor time, and device habits [17,18,19,20,21].

### 1.3. Critical Scope and Organizing Approach

Against this background, this critical narrative review appraises evidence across outdoor light, near work intensity, sleep/circadian timing, and digital device-related behavior, then derives a measurement framework. By measurement framework, we mean a structured set of exposure variables, analytic constructs, and outcome anchors that specify what to measure and how, not a causal model. It does not propose digital device use as a standalone risk factor or present the framework as a validated causal model. The central judgment is that screen duration is a nonspecific marker that may reflect indoor time, sustained near work, reduced outdoor light, educational routines, or late-day behavior.

Existing reviews and consensus reports have summarized individual behavioral risk factors. The individual exposure domains reviewed here—outdoor light, near work, sleep timing, and device behavior—are already established in prior reviews and the International Myopia Institute reports. This review therefore does not identify new risk factors. Instead, it reorganizes these known domains into a measurement-oriented, 24 h co-exposure structure (the behavioral exposures a child experiences together within a single 24 h day) with explicitly separated estimands. The focus of this review is practical. We treat device metrics as contextual markers rather than causal variables. We place the exposures within a single 24 h structure. We separate substitution, mediation, interaction, and temporal clustering as distinct questions. We propose measurement options for both older and new cohorts, and we anchor future testing to axial length where possible. The aim is to improve measurement and hypothesis testing rather than to introduce new causal claims [20,21,22,23,24,25,26,27]. Table 1 summarizes this focus.

## 2. Materials and Methods

### 2.1. Literature Identification Approach

This article is a critical narrative review supported by structured database searches used to improve transparency in source identification. The searches were not intended to establish systematic completeness, and the review was not designed as a systematic review, scoping review, mixed-method review, or quantitative evidence synthesis. We did not perform meta-analysis, PRISMA/PRISMA-ScR reporting, formal risk-of-bias appraisal, or GRADE assessment. Where we cite systematic reviews or meta-analyses, we use them only as anchor syntheses reported by other groups. PubMed/MEDLINE, Embase, Scopus, and Web of Science Core Collection were searched from inception through 5 March 2026. Search concepts combined myopia and refractive development with digital device behavior, near work and education, outdoor light exposure, and sleep/circadian timing. A targeted enrichment search identified retinal, choroidal, scleral, genetic, and omics literature relevant to axial elongation and biological plausibility. Database-specific search strings are provided in Supplementary File S1.

### 2.2. Literature Selection Approach for Narrative Synthesis

Sources were selected for narrative inclusion when they helped address one of three review questions. These concerned the strength and directness of human evidence for each exposure domain, the limitations of total screen duration, and the operationalization of a measurement framework for future studies. Priority was given to randomized or school-based outdoor interventions, prospective and objective-monitoring studies, systematic reviews and meta-analyses used as anchor syntheses, longitudinal cohorts, and studies with cycloplegic refraction or axial-length outcomes. Selected genetic, omics, and mechanistic papers were used only as an exploratory translational context.

Selection was representative and question-driven rather than exhaustive. Screening and selection were performed by three authors (J.J.P., G.E.Y., and J.K.), who judged relevance against the review questions; disagreements about inclusion were resolved by discussion until consensus. This is a purposive narrative process, not a reproducible systematic review screening protocol. For each database search, we recorded how many records were returned. These numbers appear in Supplementary File S1 only to show what the searches produced. They are not screening totals, measures of evidence strength, or proof of systematic completeness. We then read titles, abstracts, and full texts to decide whether a study was relevant to one of the exposure domains (outdoor light, near work, sleep timing, or device behavior). We also recorded how the study measured exposure and outcome, and how directly it applied to children.

### 2.3. Narrative Appraisal and Evidence Mapping Approach

For each source retained for discussion, we recorded key details when they were available: publication year, country or region, design, population, exposure definition, exposure timing, outcome definition, covariate structure, and main findings. When relevant, we also recorded viewing distance, near work continuity, device category, outdoor illuminance, sleep timing, sleep regularity, chronotype, and any co-exposure, mediation, substitution, or interaction analyses.

Evidence was interpreted narratively according to design, temporality, exposure-measurement specificity, outcome specificity, confounding susceptibility, and clinical directness. We use descriptive evidence-role language only: direct interventional or longitudinal human evidence; objective or prospective supporting evidence; cross-sectional or lower-specificity supporting evidence; and translational compatibility evidence. These roles map to three explicit evidence levels applied throughout the review. Established evidence is direct interventional or longitudinal human evidence. Supportive but interpretive evidence is objective, prospective, cross-sectional, or lower-specificity human evidence. Hypothesis-generating or speculative evidence is translational compatibility evidence. These categories are not formal risk-of-bias judgments, GRADE ratings, or pooled evidence weights [29,35,36]. The representative evidence table summarizes key human studies and their major interpretive limitations. Studies were chosen for this table when they were representative of a domain. Each also had at least one of the following features: an interventional or longitudinal design, a specific exposure or outcome definition, or a cycloplegic refraction or axial-length outcome.

### 2.4. Narrative-Review Reporting Approach

Reporting was informed by SANRA principles for narrative reviews to improve clarity of rationale, explicit aims, transparency of source identification, balanced referencing, and scientific reasoning [27]. Supplementary Table S1 provides a non-scored SANRA-informed reporting map. The database search strings are provided in Supplementary File S1 as a transparency aid.

Because this is a narrative review, the lack of a formal risk-of-bias assessment is an important limitation. To partly address it, we note design, exposure, outcome, temporality, and confounding limitations in the text and in the representative evidence table. These comments are narrative judgments and should not be read as a reproducible quality appraisal.

## 3. Critical Evidence Hierarchy and Representative Evidence

The human evidence is uneven and should be interpreted according to strength, directness, exposure specificity, and endpoint specificity. Outdoor light exposure has the strongest support for incident myopia prevention. Near work and the visual demands of schooling are important but heterogeneous. Sleep/circadian timing remains biologically plausible but has lower certainty. Digital device metrics are useful only when they clarify timing, bout structure, task context, substitution, or adherence, not when they are treated as standalone causal exposures. Table 2 summarizes the domain-level hierarchy, and Table 3 maps representative evidence to design, exposure, outcome, and limitation features.

### 3.1. Outdoor Light: Strongest Evidence for Incident Myopia Prevention

Outdoor light exposure remains the best-supported environmental domain (evidence level: established), particularly for prevention of incident myopia [10,11,12,13,14,15,37,38]. Randomized and school-based interventions support a protective effect against onset, and pooled analyses confirm a protective association for incident myopia and myopic shift. Available evidence points to a broadly dose-related benefit: more daily time outdoors and greater exposure to high ambient illuminance are associated with lower incident myopia risk. However, the exact intensity threshold, the minimum protective duration, and the most protective time of day remain uncertain [11,12,14,15,37,38]. Evidence for slowing established progression or repeated axial elongation after onset is less consistent. Interpretation should consider ancestry, urbanicity, schooling patterns, season, and access to safe outdoor environments [5,6,10,11,12,13,14,15,37,38].

### 3.2. Near Work and Education: Important but Measurement-Heterogeneous

Near work and the visual demands of schooling are supported by substantial but measurement-heterogeneous evidence (evidence level: supportive/interpretive). Previously published meta-analyses by other groups have linked greater near work to higher myopia risk, and a published meta-analysis of digital-screen exposure reports a dose-related association. We cite these existing analyses as anchor evidence; we did not perform any meta-analysis ourselves. Duration-only measures remain difficult to interpret [7,8,9,16,30,39,40,41,42,43,44]. More informative constructs include viewing distance, continuity, posture, task type, educational intensity, and time of day. Educational intensity should be interpreted as an upstream determinant that may shape outdoor displacement, sustained near work, and daily scheduling, not as evidence that leisure screen duration alone is causal [23,43,45,46,47,48,49,50,51]. Mechanistically, sustained near work is thought to act through accommodative demand and peripheral hyperopic defocus (peripheral images focusing behind the retina), both of which can drive axial elongation. Short working distance, long uninterrupted viewing, and poor posture plausibly intensify these effects [8,52,53].

### 3.3. Sleep/Circadian Timing: Biologically Plausible, Lower-Certainty Modifier

Sleep/circadian timing is best interpreted as lower-certainty modifier evidence (evidence level: supportive/interpretive, low certainty). Some reviews report weak associations of later bedtime, irregular sleep, or short sleep with myopia-related outcomes. However, effect estimates are small and inconsistent, axial-length data are sparse, and residual confounding by education, indoor time, and device use is common. These associations should be read with caution [17,18,19,20,21,54,55]. Adult evening-display experiments support biological plausibility for circadian disruption. However, pediatric myopia evidence does not establish sleep timing or evening device exposure as an independent causal domain comparable to outdoor light or near work intensity [20,21].

### 3.4. Digital Device Metrics: Contextual Measurement Marker, Not Causal Surrogate

Digital device metrics are best interpreted as contextual measurement markers (evidence status: supportive/interpretive; analytic role: contextual measurement marker). They may help identify when near work occurs and how long uninterrupted bouts last. They can also show whether activity is educational or recreational, whether exposure is concentrated in the evening, and whether intervention adherence is changing. They do not by themselves identify the biologically relevant exposure. We acknowledge that device use may also act as a behavioral exposure in its own right, for example, through sustained near viewing or evening use. We nonetheless treat the marker interpretation as our working position and as a hypothesis that future studies should test, rather than as a settled conclusion. When illuminance, viewing distance, near work continuity, and sleep timing are measured directly, device duration should be treated as a supportive context rather than as an indispensable exposure variable [7,9,20,21,28,39,56].

### 3.5. Endpoint Hierarchy: Axial Length and Cycloplegic Outcomes

Endpoint specificity is central to interpretation. Repeated axial-length measurement and cycloplegic refraction provide stronger outcome anchors than cross-sectional refractive status alone. Incident myopia is the most interpretable prevention endpoint, whereas progression and axial elongation after onset require longitudinal follow-up and treatment context. Because excessive axial growth is the pathway most directly linked to later high-myopia complications, studies with repeated axial-length outcomes should receive the greatest interpretive weight when future measurement models are tested [3,6,33,52,53,57,58].

**Table 2:** Critical evidence matrix for exposure domains and exploratory translational evidence in childhood myopia.

Evidence Domain	Narrative Evidence Role	Best-Supported Inference	Main Limitation	Unsupported Inference to Avoid
Outdoor light	Direct interventional and longitudinal human evidence; strongest for onset prevention	Greater outdoor exposure is the best-supported modifiable behavioral domain for reducing incident myopia risk	Evidence for slowing established progression or repeated axial elongation after onset is less consistent	Outdoor time alone should not be presented as a sufficient treatment for progression control.
Near work/education	Substantial but measurement-heterogeneous human evidence	Associations are more interpretable when viewing distance, continuity, educational intensity, posture, and time of day are measured	Duration-only measures are weak, and confounding by education and indoor routines is common	Screen hours alone should not be interpreted as identifying the causal near work exposure.
Sleep/circadian timing	Lower-certainty modifier evidence	Sleep timing, regularity, and evening exposure may modify risk-relevant routines and should be measured prospectively	Axial-length outcomes are sparse, and residual confounding by education, indoor time, and device use is common	Sleep timing should not be presented as an established causal domain equivalent to outdoor light or near work intensity.
Digital device behavior	Contextual measurement-marker evidence	Device metrics can help recover timing, bout structure, task context, substitution, and adherence	Total device duration is biologically nonspecific	Digital device use should not be treated as a standalone causal surrogate for myopia risk.
Omics/mechanistic evidence	Translational compatibility evidence	Mechanistic studies can support biological compatibility and generate hypotheses	Evidence is mostly indirect, exploratory, and not clinically validated	Omics evidence should not be interpreted as validating the framework or providing clinically validated biomarkers for routine use.

Table 2 note: This matrix summarizes narrative interpretation only. The evidence-role labels are descriptive and are not GRADE ratings, risk-of-bias judgments, or pooled evidence weights. Supporting references by domain: outdoor light [10,11,12,13,14,15,37,38]; near work and education [7,8,9,16,39,43,44]; sleep and circadian timing [17,18,19,20,21,54,55]; digital device behavior [7,9,28,56]; omics and mechanistic evidence [33,58,59,60,61,62].

**Table 3:** Representative human evidence informing the narrative measurement framework.

Domain	Representative Study or Synthesis	Design	Population/Evidence Source	Exposure Measurement	Outcome Definition	Main Finding	Major Interpretive Limitation
Outdoor light	He et al., 2015 [11]	Randomized clinical trial	Chinese schoolchildren	Additional outdoor time at school	Incident myopia; myopic shift	Increased school outdoor exposure reduced incident myopia	Progression effects and generalizability beyond the school setting are less certain
Outdoor light	Wu et al., 2013 [12]	School-based intervention	Taiwanese schoolchildren	Outdoor activity during class recess	Myopia onset and progression	Outdoor recess reduced new myopia and supported timetable-level prevention	School implementation and co-intervention context may limit the isolation of light-specific effects
Outdoor light	Xiong et al., 2017; Kido et al., 2024; Mei et al., 2024; Li et al., 2024 [14,15,37,38]	Systematic reviews and meta-analyses	Pooled pediatric studies and trials	Time outdoors or outdoor interventions	Incident myopia; progression; myopic shift	The protective signal is strongest for incident myopia	Exposure definitions, intervention content, and progression endpoints are heterogeneous
Objective light	Chen et al., 2024 [10]	Objective-monitoring study	Children with smartwatch-based outdoor assessment	Smartwatch-derived outdoor exposure metrics	Myopia-related outcomes	Objective outdoor metrics reduce exposure misclassification	Observational design limits causal inference
Near work	Huang et al., 2015; Dutheil et al., 2023 [16,39]	Systematic reviews and meta-analyses	Pooled pediatric near work studies	Near work duration and activity categories	Myopia or refractive outcomes	Greater near work is associated with higher myopia risk	Duration-only and questionnaire measures are heterogeneous and confounded
Near work/outdoor	Wen et al., 2020 [8]	Objective-measurement study	Children with monitored near work and outdoor exposure	Viewing distance and outdoor exposure measures	Myopia status and related outcomes	Objective viewing behavior is more informative than crude duration	Cross-sectional or limited temporal inference restricts causality
Combined exposure patterns	Lanca et al., 2022 [7]	Consortium analysis	Schoolchildren across the consortium datasets	Near work, screen time, and outdoor time	Myopia in schoolchildren	Screen time behaves as a partial marker within a broader behavioral ecology	Harmonized exposure variables remain imperfect proxies
Digital device exposure	Ha et al., 2025 [9]	Systematic review and dose–response meta-analysis	Pooled studies of screen exposure and myopia	Digital screen duration	Myopia and myopic shift	Screen duration shows a dose-related association	Duration does not identify viewing distance, light context, timing, or substitution
Education	Cuellar-Partida et al., 2016 [23]	Mendelian randomization	Genetic epidemiology of educational attainment and refractive outcomes	Education-related genetic instruments	Refractive error or myopia	Educational attainment has causal relevance to refractive outcomes	Mediating behavioral components and pleiotropy requires cautious interpretation
Education/displacement	Clark et al., 2023 [43]	Observational mediation-oriented analysis	Population-based myopia data	Education and outdoor time	Myopia-related outcomes	Time outdoors partly accounts for the education-related signal	Residual confounding and pathway assumptions remain possible
Sleep/circadian timing	Liu et al., 2023; Jin et al., 2024; Zhao et al., 2024 [17,18,19]	Systematic reviews and meta-analyses	Children and adolescents	Sleep duration, timing, and regularity	Myopia-related outcomes	Sleep timing and insufficiency show modest associations	Axial-length data are sparse, and confounding by education/device routines is common
Evening display timing	Chang et al., 2015; Cajochen et al., 2011 [20,21]	Controlled adult experiments	Adult experimental participants	Evening light-emitting display exposure	Melatonin, circadian timing, alertness	Late display exposure has biologically distinct circadian effects	Adult sleep physiology cannot be extrapolated as direct pediatric myopia evidence

## 4. Measurement Framework Derived from the Evidence

The measurement framework follows from the evidence hierarchy summarized above. It separates directly measured exposure domains from lower-certainty modifiers and contextual measurement markers. This structure is intended to improve exposure measurement and hypothesis testing, not to assert a validated causal model.

### 4.1. Primary Exposure Domains: Outdoor Light and near Work Intensity

Outdoor light and near work intensity should form the minimum exposure core. Outdoor exposure should be measured as daylight or eye-level illuminance whenever possible, not only as reported time outdoors. Near work intensity should capture viewing distance, continuity, task type, educational load, posture, and time of day rather than total duration alone.

### 4.2. Lower-Certainty Modifier: Sleep/Circadian Timing

Sleep/circadian timing should be measured as a lower-certainty modifier. Relevant variables include sleep duration, bedtime, timing variability, chronotype, and evening exposure under low ambient light. These variables should not be treated as established primary causal exposures, but they may help test whether the timing of near work and light exposure modifies ocular-growth outcomes.

### 4.3. Contextual Marker: Digital Device-Related Behavior

Digital device-related behavior should be recorded when it adds information about timing, bout structure, task context, substitution, or adherence. A device-based measurement approach is useful only when it clarifies how established exposure domains occur within daily routines. For example, device timestamps can show whether near work is concentrated in the evening. Device logs can reveal whether a reduction in screen time is replaced by outdoor time (substitution) rather than by other indoor near work. Total screen duration should not be treated as biologically coherent exposure.

### 4.4. Higher-Order Constructs: Substitution, Mediation, Interaction, and Temporal Clustering

Substitution asks what activity is replaced within a fixed 24 h day. Mediation asks whether one exposure partly transmits the effect of another. Interaction or effect modification asks whether the association of one exposure differs across levels of another. Temporal clustering asks whether low outdoor light, sustained near work, and delayed or irregular sleep occur together within the same child, day, or school routine. These constructs should be modeled explicitly rather than inferred from co-occurrence alone.

### 4.5. Outcome Anchor: Axial Elongation

**Figure 1:**
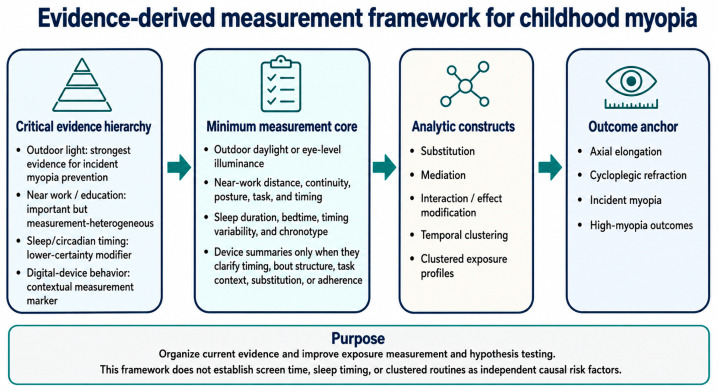
Evidence-derived measurement framework for childhood myopia. This figure was generated using an AI tool (ChatGPT).

When feasible, studies should use axial elongation as a common primary outcome, so that different cohorts can be compared using the same measure of eye growth. Cycloplegic refraction and incident myopia classification should support this outcome. Cross-sectional refractive status can be useful but is less specific. For legacy cohorts, questionnaire-based proxies can still support harmonized analyses when quality flags are stated explicitly. Figure 1 provides a high-level measurement-priority summary derived from the evidence hierarchy; operational definitions and detailed 24 h constructs are provided in Supplementary Table S2.

The figure summarizes exposure-measurement priorities, analytic constructs, and axial-growth outcome anchors in a conceptual format. It does not present a detailed 24 h causal diagram, a validated causal model, or a formal DAG. The four elements shown—critical evidence hierarchy, minimum measurement core, analytic constructs, and outcome anchor—are intended to guide what future studies should measure and test. The specific temporal relationships among outdoor light exposure, near work, sleep/circadian timing, digital device behavior, and ocular growth should be specified in study-specific causal diagrams.

## 5. Translational Compatibility: Exploratory Mechanistic Evidence

Mechanistic, genetic, and omics findings are retained as a focused translational compatibility check, not as a pillar of the causal framework (evidence level: hypothesis-generating/compatibility). They support biological plausibility for links among light exposure, near viewing behavior, circadian timing, ocular tissues, and axial elongation. However, they do not validate device-related exposure metrics, provide clinically deployable biomarkers, or replace human epidemiologic and interventional evidence. Two mechanisms are commonly proposed. Bright outdoor light is thought to increase retinal dopamine release, which slows axial elongation, and this light-responsive signaling helps explain the protective effect of time outdoors [53,57,63]. In parallel, ocular growth follows circadian rhythms in the retina and choroid, so the timing of light and near work, not only their total amount, may influence how the eye grows [54,58,63,64].

Genetic susceptibility contributes substantially to myopia. Genome-wide association studies have identified many risk loci, and polygenic risk scores capture part of the inherited liability [24,25,47,50,51]. Mendelian randomization using education-related genetic instruments supports a causal role for schooling in refractive development [23]. Genetic liability is best understood as a time-invariant modifier: it does not change with daily behavior, but it may change how strongly behavioral exposures affect eye growth. The framework therefore treats genetic liability as a stratifying modifier across the whole exposure structure, not as an interchangeable behavioral exposure. Multi-omic and dynamic tissue findings remain exploratory and are best used for hypothesis generation and nested enrichment studies, not as routine biomarkers [58,59,60,61,62,64,65,66,67,68,69,70]. Because dynamic biomarkers may vary with clock time and recent exposure, future studies should document sampling time and recent light, near work, and sleep history. Prospective endpoint testing should align with established axial-length and cycloplegic refraction instrumentation standards [34]. Supplementary Figure S1 and Supplementary Table S3 summarize this mechanistic compatibility bridge.

## 6. Integrated Interpretation and Methodological Implications

### 6.1. Reconciling Inconsistencies Across Studies

Mixed findings across the screen-exposure literature are expected when exposure definition is underspecified. Questionnaire screen duration does not capture viewing distance, continuity, ambient illuminance, task context, or evening concentration, and may therefore misclassify biologically distinct routines [8,9,28,30,39,40]. Confounding by education intensity, urbanicity, socioeconomic context, parental myopia, season, and baseline refractive status remains common. Reverse causation is also plausible because children with emerging myopia may shift toward indoor near activities. Interpretation is strongest when prospective studies combine objective exposure measurement with axial length and cycloplegic refraction [8,10,30,40,41,42].

### 6.2. Supported Inferences Versus Hypothesis-Generating Links

The supported inference is narrow: greater outdoor exposure is the best-established modifiable behavioral domain for reducing incident myopia [11,12,13,14,15,37,38]. Near work intensity and educational demand remain important but require better measurement of viewing distance, continuity, timing, and task context [7,8,16,23,39,43]. Sleep/circadian timing and omics findings are hypothesis-generating modifiers or compatibility evidence, not clinically validated causal domains. Device-based summaries are most useful when they recover time-structured behavior beyond directly measured light, near viewing, and sleep variables [20,21,28].

### 6.3. Methodological Implications

Future analyses should prespecify the estimand before assigning analytic roles to education, device use, outdoor exposure, sleep, or near work. Standard confounder adjustment remains necessary, but substitution, mediation, interaction, and clustered exposure profiles answer different questions and require different models [28,29,71,72]. Causal diagrams should define whether device use is a proxy, mediator, bundled exposure summary, or practical intervention target for the specific question. Objective measurement of illuminance, viewing distance, device timestamps, and sleep timing is preferred when feasible, with privacy-preserving aggregation of device logs [8,10,30,40,41,42,73,74]. Multiple-exposure and exposome (the full set of environmental exposures a person experiences) frameworks can help organize coupled daily exposures [75,76]. Harmonization across legacy cohorts should use transparent crosswalks and quality flags rather than assuming equivalence between questionnaire proxies and objective measurements [28,31,32].

## 7. Future Testing Priorities and Study Designs

### 7.1. Tiered Measurement Framework for Prospective Testing

Prospective testing should use a concise tiered framework rather than an expansive minimum dataset. The three measurement tiers defined in Section 4 (legacy-feasible, prospective-standard, and optional enrichment) apply directly to prospective testing [77,78,79]. Pubertal stage should be recorded when it plausibly shapes sleep timing or outdoor opportunity [80]. Supplementary Figure S2 summarizes these tiers.

### 7.2. Priority Hypotheses for Future Studies

The framework suggests a limited set of testable hypotheses for future research.

Priority hypotheses should test several specific questions. Does timing and bout structure (how long and how often an activity occurs) add information beyond total duration? Does outdoor exposure weaken the link between intensive near work and ocular growth? Do sleep or circadian timing change near work associations? Do clustered exposure profiles replicate across cohorts? Does genetic liability change how strongly environmental exposures act? Evidence relevant to these questions spans behavioral, sleep, genetic, and device studies [9,10,20,21,24,25,28,43,44,45,46,47,48,49,50,51,56]. Mechanistic and omics hypotheses should be evaluated only as exploratory links to axial-growth velocity until prospectively replicated [22,33,59,60,61,62].

### 7.3. Study Designs and Implementation Priorities

#### 7.3.1. Prospective Cohort with Objective Monitoring

A prospective cohort combining wearable light sensing, near viewing metrics, actigraphy-derived sleep timing, and privacy-preserving device summaries is the most direct design for testing substitution, interaction, and temporal clustering [8,10,28,30,40,41,42,73,74]. Primary endpoints should prioritize axial-length change and cycloplegic refraction, with education intensity and device metrics assigned roles in a prespecified causal diagram. Advances in wearable sensing and computational analysis point to relevant technical directions. One wearable multi-sensor system uses language-model processing for continuous signal decoding [81]. The PEGSYS platform combines portable fundus imaging with cloud-based AI for glaucoma screening and grading [82]. These examples are drawn from other application domains and are not evidence in myopia; they are cited only to illustrate possible directions for multimodal sensing, image analysis, and mobile/cloud deployment.

#### 7.3.2. School-Level Intervention Package

A pragmatic school-level package that increases protected outdoor time, structures near work breaks, and reduces evening device concentration should be viewed as a candidate test of the framework, not as a strategy already proven superior. Devices function mainly as practical handles for modifying routines across coupled domains, not as singular causal agents [11,12,14,15,16,20,21,37,38,39,40].

#### 7.3.3. Optional Mechanistic and Implementation Extensions

Nested mechanistic substudies can test whether retinal or choroidal imaging markers, selected biomarkers, or genetic stratification align with axial-growth velocity more closely than questionnaire exposure summaries [24,25,46,47,50,51,58,59,61,62,63,65,66]. Health-economic evaluation can be retained as a secondary implementation extension when school-level prevention strategies are tested at scale [1,2,3,4,83,84]. Supplementary Table S4 maps operational constructs to measures, designs, endpoints, and falsifiable results. The extended representative study inventory is retained in Supplementary Table S5, and the plain-language glossary is retained in Supplementary Table S6.

## 8. Clinical and Public-Health Implications

### 8.1. Clinical Implications: Onset Prevention Versus Progression Control

This framework does not establish new clinical causal claims. It organizes current evidence so that counseling can separate prevention of myopia onset from control of established progression and can avoid treating crude screen duration as the main clinical target.

For children who are not yet myopic, increasing protected outdoor light exposure remains the best-supported behavioral strategy for reducing incident myopia risk [11,12,13,14,15,37,38]. For children with established myopia, behavioral optimization should complement, not replace, validated progression-control interventions and longitudinal ocular-growth monitoring [34,78,79,85,86,87,88]. Axial-length monitoring provides the most coherent clinical bridge because it tracks the biological growth pathway linked to later high-myopia complications [3,6,33,34,74,78,79]. Pandemic home-confinement studies are retained only as contextual evidence of co-occurring routine shifts rather than screen-specific causation [89,90].

Clinical counseling can therefore move beyond generic screen restriction toward specific routine-level questions: Is daylight exposure protected? Are near work bouts long and uninterrupted? Is the working distance short? Is heavy near work concentrated in the evening? Are sleep timing and school routines stable enough to support healthier visual behavior [5,7,8,9,16,17,18,19,20,21,28]?

### 8.2. Public-Health, School-Based Implementation, and Equity

At the public-health level, the strongest implication is to protect school- and community-level outdoor opportunity, especially for onset prevention [11,12,13,14,15,37,38]. Packages that combine protected outdoor time with healthier near work structure and less evening concentration on device-based schoolwork are reasonable candidates for future testing. However, they should be described as evidence-informed implementation strategies rather than proven screen-specific interventions.

Equity is central because educational pressure, urban housing, school schedules, and safe outdoor access differ substantially across populations [77,83,84]. Prevention strategies that rely only on household behavior change may widen disparities when structural opportunities for outdoor activity are absent. The framework is therefore intended to support system-level routines that make healthier visual exposure patterns feasible, not to blame individual device use.

## 9. Limitations

### 9.1. Limitations Inherent to Narrative Synthesis and Heterogeneous Exposure Definitions

This critical narrative review does not provide exhaustive capture, formal risk-of-bias appraisal, or quantitative pooling. Source selection was representative and question-driven, and evidence strength was interpreted narratively from design, exposure-measurement specificity, outcome specificity, temporality, confounding susceptibility, and clinical directness. Because selection was purposive and depended on the authors’ judgment, it introduces selection bias, and the authors’ interpretation may have influenced the resulting framework. The descriptive evidence-role categories and major-limitation comments in Table 3 are interpretive aids, not GRADE ratings, formal risk-of-bias judgments, or pooled evidence weights. Exposure definitions remain heterogeneous, especially for device-related behavior, and English-language full-text selection may have introduced language bias.

### 9.2. Evidence Gaps and Generalizability Limits

Important gaps remain. Most studies measure only one or two domains rather than outdoor light, near viewing, sleep timing, device context, and axial growth within the same cohort [8,10,30,40,41,42,73]. Formal tests of substitution, mediation, interaction, and temporal clustering are therefore limited. Omics and mechanistic studies are exploratory and may not generalize to early pediatric myopia development [59,60,61,62]. The proposed framework has not been prospectively validated against simpler models and should be tested directly before being used for prediction, treatment selection, or clinical risk stratification. Much of the strongest evidence, including the school-based outdoor-time trials, comes from East Asian school-aged populations (for example, China and Taiwan). Ancestry, urban environment, schooling intensity, and safe outdoor access differ across regions. The framework is therefore most directly supported in East Asian educational settings, and its use in other populations should be validated prospectively.

## 10. Conclusions

Total screen duration is not a sufficient exposure measure for childhood myopia research. It does not capture daylight displacement, viewing distance, near work continuity, task context, timing, or sleep and circadian setting. Across the current evidence, outdoor light has the strongest support for preventing myopia onset. Near work and educational demand matter, but are measured inconsistently. Sleep and circadian timing are lower-certainty modifiers, and digital device behavior is best treated as a contextual marker [5,7,8,9,10,11,12,13,14,15,16,17,18,19,20,21,28,37,38,39]. The framework turns this hierarchy into practical steps: tiered exposure measurement; explicit analysis of substitution, mediation, interaction, and temporal clustering; and axial elongation as the preferred common outcome. Its aim is to improve measurement and prospective testing, not to claim that device use, sleep timing, or clustered routines independently cause myopia.

## Figures and Tables

**Table 1:** Scope of this review relative to prior behavioral myopia reviews and consensus reports.

Dimension	Common Emphasis in Prior Reviews or Consensus Reports	Operational Focus of This Framework
Exposure concept	Individual behavioral risk-factor categories	A 24 h co-exposure structure linking outdoor light, near work, sleep timing, and device context
Digital device use	Often summarized as total screen duration	Contextual marker of timing, bout structure, task context, substitution, and adherence
Timing and bout structure	Usually secondary or implicit	Specified as separate estimands from the total duration
Analytic focus	Single-exposure models with confounder adjustment	Separate tests of substitution, mediation, interaction, and temporal clustering
Measurement strategy	Study-specific or heterogeneous measures	Tiered legacy-feasible, prospective-standard, and optional enrichment measures with quality flags
Outcome anchor	Mixed refractive endpoints	Axial elongation prioritized where feasible, supported by cycloplegic refraction and incident myopia classification

Table 1 note: supporting references for the emphases summarized above: individual behavioral risk-factor and consensus framing [5,6]; screen-duration summaries [7,9]; timing and bout structure [7,28]; single-exposure analytic models [28,29]; heterogeneous measurement and harmonization [30,31,32]; mixed refractive versus axial endpoints [33,34].

## Data Availability

No new participant-level datasets, unpublished datasets, or custom code were generated or analyzed in the current study. Database search strings, the SANRA-informed reporting map, operational definitions, extended representative-study inventory, and glossary are provided in the Supplementary Materials.

## Supplementary Materials

The following supporting information can be downloaded at: https://www.mdpi.com/article/10.3390/life16071178/s1, Supplementary File S1: Database search strings and narrative source-identification approach; Figure S1: Mechanistic compatibility bridge; Figure S2: Measurement tiers and future-study priorities; Table S1: SANRA-informed reporting map for the narrative review; Table S2: Operational definitions and measurement constructs for the primary exposure domains, lower-certainty modifier, and higher-order exposure structure; Table S3: Candidate bridge pathways and exploratory mechanistic evidence across the primary exposure domains, lower-certainty modifier, and ocular tissues; Table S4: Mapping of operational definitions and future-study hypotheses to measures, study designs, and endpoints; Table S5: Extended representative human-study inventory across exposure domains and clustered exposure patterns; Table S6: Plain-language glossary of measurement and analytic terms.

## Abbreviations

The following abbreviations are used in this manuscript:

AL	Axial length
CCT	Correlated color temperature
COVID-19	Coronavirus disease 2019
DAG	Directed acyclic graph
DIMS	Defocus incorporated multiple segments
DNA	Deoxyribonucleic acid
ECM	Extracellular matrix
GWAS	Genome-wide association study
HAL	Highly aspherical lenslet
HM	High myopia
IMI	International Myopia Institute
IOL	Intraocular lens
miRNA	MicroRNA
OCT	Optical coherence tomography
PRISMA	Preferred Reporting Items for Systematic Reviews and Meta-Analyses
PRS	Polygenic risk score
RLRL	Repeated low-level red light
ROS	Reactive oxygen species
SANRA	Scale for the Assessment of Narrative Review Articles
SE	Spherical equivalent
