# Supplementary material for: Beyond Screen Time: A Measurement Framework for Behavioral Exposures in Childhood Myopia
Source: Life (Basel). 2026 Jul 16;16(7):1178. doi: 10.3390/life16071178 (PMC13413134; doi:10.3390/life16071178)
Supplement: Supplementary file 1 [file life-16-01178-s001.zip › life-4418082-supplementary.pdf]

Supplementary Materials

Supplementary File S1. Database search strings and narrative source-identification approach

Searches were designed to support a critical narrative review with transparent source identification. They were not designed to establish systematic completeness, PRISMA-style inclusion, formal screening denominators, or quantitative evidence weighting. Searches were conducted from database inception to March 5, 2026, without database-level date restrictions. English-language selection was applied during narrative full-text review rather than as a database filter. Module A targeted epidemiologic and interventional evidence by combining terms for myopia and refractive development with terms for digital-device behavior, near work and educational intensity, outdoor light exposure, and sleep or circadian characteristics. Module B targeted mechanistic and translational enrichment for retinal, choroidal, scleral, DNA-based genetic, and dynamic omics evidence relevant to axial elongation.

Search strings were paired with backward citation checking of anchor reviews, meta-analyses, consensus reports, and landmark primary studies. The retrieval counts listed below describe database search output only. They are retained to document search breadth and should not be interpreted as screening denominators, inclusion counts, formal evidence weights, or a claim of systematic-review completeness. Source selection followed a purposive, question-driven process. Three authors (J.J.P., G.E.Y., and J.K.) screened titles, abstracts, and full texts and judged each study's relevance to the review questions, its exposure and outcome specificity, and its clinical directness; disagreements were resolved by discussion until consensus. Priority was given to randomized or school-based outdoor interventions, prospective or objective-monitoring studies, systematic reviews and meta-analyses used as anchor syntheses, and studies reporting cycloplegic refraction or axial length. This process was designed for transparency of source identification and is not a reproducible systematic-review screening protocol.

PubMed/MEDLINE

Module A. Core epidemiologic and interventional search

N = 3,104

("Myopia"[Mesh] OR myopia[tiab] OR myopic[tiab] OR "refractive error*"[tiab] OR "axial length"[tiab] OR "axial elongation"[tiab] OR "high myopia"[tiab] OR "pathologic myopia"[tiab])
AND ("Computers"[Mesh] OR "Cell Phone"[Mesh] OR "Internet"[Mesh] OR "Video Games"[Mesh] OR "Reading"[Mesh] OR "Education"[Mesh] OR "Students"[Mesh] OR "Outdoor Recreation"[Mesh] OR "Sunlight"[Mesh] OR "Light"[Mesh] OR "Sleep"[Mesh] OR "Circadian Rhythm"[Mesh] OR smartphone*[tiab] OR "mobile phone*"[tiab] OR tablet*[tiab] OR computer*[tiab] OR screen*[tiab] OR "screen time"[tiab] OR "digital device*"[tiab] OR "electronic device*"[tiab] OR "video display terminal*"[tiab] OR "online learning"[tiab] OR "remote learning"[tiab] OR elearning[tiab] OR "e learning"[tiab] OR "near work"[tiab] OR reading[tiab] OR homework[tiab] OR studying[tiab] OR education*[tiab] OR "study time"[tiab] OR "educational intensity"[tiab] OR outdoor*[tiab] OR illuminance[tiab] OR daylight[tiab] OR sunlight[tiab] OR sleep[tiab] OR bedtime[tiab] OR chronotype[tiab] OR circadian[tiab] OR melatonin[tiab] OR "sleep duration"[tiab] OR "sleep regularity"[tiab])
AND ("Child"[Mesh] OR "Adolescent"[Mesh] OR child*[tiab] OR adolescen*[tiab] OR teen*[tiab] OR youth[tiab] OR pediatric*[tiab] OR paediatric*[tiab] OR schoolchild*[tiab] OR student*[tiab])
NOT (animals[mh] NOT humans[mh])

Module B. Mechanistic and omics enrichment search

N = 9,737 (6,167 + 3,570)

("Myopia"[Mesh] OR myopia[tiab] OR myopic[tiab] OR "refractive error*"[tiab] OR "axial length"[tiab] OR "axial elongation"[tiab] OR "high myopia"[tiab] OR "pathologic myopia"[tiab])
AND (retina*[tiab] OR retinal[tiab] OR choroid*[tiab] OR sclera*[tiab] OR "extracellular matrix"[tiab] OR collagen[tiab] OR dopamine[tiab] OR melatonin[tiab] OR circadian[tiab] OR genetics[tiab] OR genotype*[tiab] OR "gene environment"[tiab] OR "polygenic risk score*"[tiab] OR "genome wide association"[tiab] OR GWAS[tiab] OR "Mendelian randomization"[tiab] OR transcriptom*[tiab] OR proteom*[tiab] OR metabolom*[tiab] OR epigenom*[tiab] OR biomarker*[tiab])

Embase

Module A. Core epidemiologic and interventional search

N = 4,444

('myopia'/exp OR myopia:ti,ab,kw OR myopic:ti,ab,kw OR 'refractive error*':ti,ab,kw OR 'axial length':ti,ab,kw OR 'axial elongation':ti,ab,kw OR 'high myopia':ti,ab,kw OR 'pathologic myopia':ti,ab,kw)
AND (smartphone*:ti,ab,kw OR 'mobile phone*':ti,ab,kw OR tablet*:ti,ab,kw OR computer*:ti,ab,kw OR screen*:ti,ab,kw OR 'screen time':ti,ab,kw OR 'digital device*':ti,ab,kw OR 'electronic device*':ti,ab,kw OR 'video display terminal*':ti,ab,kw OR 'online learning':ti,ab,kw OR 'remote learning':ti,ab,kw OR elearning:ti,ab,kw OR 'e learning':ti,ab,kw OR 'near work':ti,ab,kw OR reading:ti,ab,kw OR homework:ti,ab,kw OR studying:ti,ab,kw OR education*:ti,ab,kw OR 'study time':ti,ab,kw OR 'educational intensity':ti,ab,kw OR outdoor*:ti,ab,kw OR illuminance:ti,ab,kw OR daylight:ti,ab,kw OR sunlight:ti,ab,kw OR sleep:ti,ab,kw OR bedtime:ti,ab,kw OR chronotype:ti,ab,kw OR circadian:ti,ab,kw OR melatonin:ti,ab,kw OR 'sleep duration':ti,ab,kw OR 'sleep regularity':ti,ab,kw)
AND ('child'/exp OR 'adolescent'/exp OR child*:ti,ab,kw OR adolescen*:ti,ab,kw OR teen*:ti,ab,kw OR youth:ti,ab,kw OR pediatric*:ti,ab,kw OR paediatric*:ti,ab,kw OR schoolchild*:ti,ab,kw OR student*:ti,ab,kw)
NOT ([animals]/lim NOT [humans]/lim)

Module B. Mechanistic and omics enrichment search

N = 16,562

('myopia'/exp OR myopia:ti,ab,kw OR myopic:ti,ab,kw OR 'refractive error*':ti,ab,kw OR 'axial length':ti,ab,kw OR 'axial elongation':ti,ab,kw OR 'high myopia':ti,ab,kw OR 'pathologic myopia':ti,ab,kw)
AND (retina*:ti,ab,kw OR retinal:ti,ab,kw OR choroid*:ti,ab,kw OR sclera*:ti,ab,kw OR 'extracellular matrix':ti,ab,kw OR collagen:ti,ab,kw OR dopamine:ti,ab,kw OR melatonin:ti,ab,kw OR circadian:ti,ab,kw OR genetics:ti,ab,kw OR genotype*:ti,ab,kw OR 'gene environment':ti,ab,kw OR 'polygenic risk score*':ti,ab,kw OR 'genome wide association':ti,ab,kw OR GWAS:ti,ab,kw OR 'Mendelian randomization':ti,ab,kw OR transcriptom*:ti,ab,kw OR proteom*:ti,ab,kw OR metabolom*:ti,ab,kw OR epigenom*:ti,ab,kw OR biomarker*:ti,ab,kw)

Scopus

Module A. Core epidemiologic and interventional search

N = 4,820

TITLE-ABS-KEY((myopia OR myopic OR "refractive error*" OR "axial length" OR "axial elongation" OR "high myopia" OR "pathologic myopia") AND (smartphone* OR "mobile phone*" OR tablet* OR computer* OR screen* OR "screen time" OR "digital device*" OR "electronic device*" OR "video display terminal*" OR "online

learning" OR "remote learning" OR elearning OR "e learning" OR "near work" OR reading OR homework OR studying OR education* OR "study time" OR "educational intensity" OR outdoor* OR illuminance OR daylight OR sunlight OR sleep OR bedtime OR chronotype OR circadian OR melatonin OR "sleep duration" OR "sleep regularity") AND (child* OR adolescen* OR teen* OR youth OR pediatric* OR paediatric* OR schoolchild* OR student*))
AND NOT TITLE-ABS-KEY("animal model*" OR mouse OR mice OR rat OR rats OR rabbit OR rabbits OR chick OR chicks)

Module B. Mechanistic and omics enrichment search

N = 18,020

TITLE-ABS-KEY((myopia OR myopic OR "refractive error*" OR "axial length" OR "axial elongation" OR "high myopia" OR "pathologic myopia") AND (retina* OR retinal OR choroid* OR sclera* OR "extracellular matrix" OR collagen OR dopamine OR melatonin OR circadian OR genetics OR genotype* OR "gene environment" OR "polygenic risk score*" OR "genome wide association" OR GWAS OR "Mendelian randomization" OR transcriptom* OR proteom* OR metabolom* OR epigenom* OR biomarker*))

Web of Science Core Collection

Module A. Core epidemiologic and interventional search

N = 8,284

TS=((myopia OR myopic OR "refractive error*" OR "axial length" OR "axial elongation" OR "high myopia" OR "pathologic myopia") AND (smartphone* OR "mobile phone*" OR tablet* OR computer* OR screen* OR "screen time" OR "digital device*" OR "electronic device*" OR "video display terminal*" OR "online learning" OR "remote learning" OR elearning OR "e learning" OR "near work" OR reading OR homework OR studying OR education* OR "study time" OR "educational intensity" OR outdoor* OR illuminance OR daylight OR sunlight OR sleep OR bedtime OR chronotype OR circadian OR melatonin OR "sleep duration" OR "sleep regularity") AND (child* OR adolescen* OR teen* OR youth OR pediatric* OR paediatric* OR schoolchild* OR student*))
NOT TS=("animal model*" OR mouse OR mice OR rat OR rats OR rabbit OR rabbits OR chick OR chicks)

Module B. Mechanistic and omics enrichment search

N = 13,556

TS=((myopia OR myopic OR "refractive error*" OR "axial length" OR "axial elongation" OR "high myopia" OR "pathologic myopia") AND (retina* OR retinal OR choroid* OR sclera* OR "extracellular matrix" OR collagen OR dopamine OR melatonin OR circadian OR genetics OR genotype* OR "gene environment" OR "polygenic risk score*" OR "genome wide association" OR GWAS OR "Mendelian randomization" OR transcriptom* OR proteom* OR metabolom* OR epigenom* OR biomarker*))

Supplementary Tables

Supplementary Table S1. SANRA-informed reporting map for the narrative review

Maps each SANRA reporting item to its location in the revised manuscript, documenting reporting transparency for this narrative review.

SANRA item	Revised manuscript location	Use in revised manuscript	Comment
Justification of the article's importance for the readership	Sections 1.1 to 1.3; Box 1	Addressed	Disease burden, clinical significance, conceptual gap, and rationale for an exposure-measurement framework are stated.
Statement of concrete aims or formulation of questions	Section 1.3; Box 1	Addressed	Aims distinguish critical narrative appraisal from the downstream measurement framework.
Description of literature search	Sections 2.1 and 2.2; Supplementary File S1	Addressed	Information sources, search date, database-specific queries, and backward citation checking are specified as source-identification aids, not systematic-review denominators.
Referencing	Throughout manuscript	Addressed	Recent peer-reviewed sources and landmark articles are prioritized across epidemiologic, interventional, measurement, and translational domains.
Scientific reasoning	Sections 3 to 10	Addressed	Reasoning proceeds from evidence hierarchy to representative evidence mapping, measurement framework, implications, limitations, and conclusions.
Appropriate presentation of data	Table 1; Table 2; Figure 1; Supplementary Tables S2-S6; Supplementary Figures S1-S2	Addressed	The revised manuscript provides a main-text representative evidence table, treats Figure 1 as a high-level measurement-priority summary rather than a formal causal diagram, and avoids formal risk-of-bias grading, GRADE ratings, or quantitative pooling.

Supplementary Table S2. Operational definitions and measurement constructs for the primary exposure domains, lower-certainty modifier, and higher-order exposure structure

Provides structured short-form operational definitions, legacy-cohort crosswalk variables, and concise analytic cues for alignment across epidemiologic, interventional, and translational studies.

Level or construct	Operational definition	Legacy-feasible proxy or crosswalk	Preferred objective or prospective measure	Primary analytic role or endpoint linkage
Foundational measured variable				
Digital-device contextual measurement marker	Device use can mark the timing and context of near work during the day. In the evening, device use may also indicate light exposure near sleep onset under low ambient light.	Daily device duration by school-day versus weekend; share occurring in the pre-sleep or evening window.	Timestamped device summaries by time window, device type, longest continuous bout, and where feasible light context or bedtime-/phase-anchored windows.	Timing/substitution/clustering anchor; evening-window metrics may also index direct circadian-light input; not a standalone cause.
Primary exposure domains and modifier				
Primary domain 1. Outdoor light exposure	Reduced exposure to outdoor daylight and eye-level illuminance, including diminished access to high-illuminance periods.	Time outdoors categories, recess duration, season-adjusted outdoor proxy.	Wearable light sensor data, time above prespecified lux thresholds, daylight episode counts, and where feasible spectral context or CCT.	Primary prevention domain; strongest for incident myopia; pair with cycloplegic SE and AL.
Primary domain 2. Near-work intensity	Sustained near viewing characterized by short working distance, continuity, task density, and school-related visual load.	Homework hours, reading time, reported viewing distance category, longest self-reported continuous near work bout.	Objective viewing distance sensors, task timestamps, longest uninterrupted bout, break frequency, and task-specific device-use summaries.	Main visual-demand domain; test timing and interaction with daylight and sleep.

Level or construct	Operational definition	Legacy-feasible proxy or crosswalk	Preferred objective or prospective measure	Primary analytic role or endpoint linkage
Lower-certainty modifier. Sleep/circadian timing	Short sleep, delayed or irregular timing, chronotype mismatch, and evening display exposure in biologically vulnerable pre-sleep windows.	Bedtime, wake time, weekday-weekend timing difference, self-reported evening screen concentration.	Actigraphy or validated sleep tracking, time-stamped evening device exposure, and where feasible circadian biomarkers collected with documented clock time or phase anchoring.	Lower-certainty modifier; interpret more cautiously than outdoor light and near-work intensity.
24-hour daily exposure pattern				
Timing	Clock time at which near work, device use, outdoor exposure, and sleep-related behaviors occur.	Morning, after-school, and evening exposure windows derived from questionnaires or diaries.	Continuous timestamped logs aligned with illuminance and sleep timing.	Compare equal-duration exposures that occur at different times of day.
Continuity	Whether near work is accumulated in long uninterrupted bouts or dispersed with breaks.	Longest reported study bout; break frequency category.	Sensor-derived bout length, break interval, and continuous near viewing time.	Test whether bout structure predicts AL change beyond total duration.
Sequence	Ordering of exposures within a day, such as outdoor time before or after sustained near work.	Diary-based before-school or after-school outdoor placement.	Ordered event streams linking daylight, near work, and sleep windows.	Supports lagged or sequence-aware models, not isolated daily totals.
Temporal clustering	Co-accumulation of multiple risk-relevant exposures within the same daily window, especially in the evening.	Evening cluster score combining low daylight, high near work, and delayed bedtime proxies.	Integrated multimodal time stamps from wearables and devices.	Defines higher-risk daily patterns despite similar total exposure duration.
Higher-order analytic constructs				
Substitution or displacement	Counterfactual activity replaced when screen-related time increases within a fixed day.	Crosswalks for screen for outdoor, screen for sleep, and screen for non-screen near work replacement.	Compositional time-use framework using directly measured daylight, near work, sleep, and device timestamps.	Analyze as reallocation within a fixed 24-hour day; do not infer from screen duration alone.

Level or construct	Operational definition	Legacy-feasible proxy or crosswalk	Preferred objective or prospective measure	Primary analytic role or endpoint linkage
Interaction or effect modification	Whether the association of one exposure domain changes across levels of another exposure or inherited susceptibility.	Combined exposure categories such as high near work with low outdoor time, or high PRS with high exposure load.	Explicit interaction terms on additive or multiplicative scales, with prespecified strata.	Distinguish buffering or amplification from parallel main effects.
Mediation	Partial transmission of one exposure effect through another pathway or behavior.	Education intensity to outdoor time or near work continuity crosswalks.	Prospective mediation models with confounder structure appropriate to the target pathway.	Separate from substitution and interaction; do not infer from co-occurrence alone.
Clustered exposure profiles	Clustered profiles of low daylight, long continuous near work, and irregular sleep timing within persons or days.	Latent class or rule-based profiles reconstructed from harmonized legacy variables.	Multimodal clustering from device, light, sleep, and questionnaire data.	Descriptive or predictive layer only; direct empirical support remains limited.
Core outcomes and context				
Minimum outcome core	Preferred shared growth endpoint is axial elongation, supported by cycloplegic refraction and incident myopia classification.	Baseline and follow-up cycloplegic SE category; incident myopia status where biometry is unavailable.	Standardized cycloplegic SE, repeated AL biometry, and where feasible growth velocity over follow-up.	AL is the preferred shared endpoint linking upstream exposure to downstream high-myopia risk.
Minimum context core	Upstream and baseline contextual drivers that shape the exposure framework and outcome trajectory rather than constituting exposure-domain components.	Chronological age, sex, education intensity, season, urbanicity, ethnicity or ancestry, socioeconomic status, household routines, parental myopia, pubertal stage, baseline refractive status or axial length, and myopia-control treatment exposure.	Standardized covariates from registry, survey, school schedule, geographic context, pubertal assessment, baseline biometry, and treatment records.	Required for confounding control, harmonization, treatment stratification, and cross-cohort interpretation.

Level or construct	Operational definition	Legacy-feasible proxy or crosswalk	Preferred objective or prospective measure	Primary analytic role or endpoint linkage
Optional biologic enrichment	Subset-level measures intended to test mechanism and heterogeneity rather than define the minimum dataset; includes time-invariant genetics and dynamic biomarkers with different pre-analytic requirements.	None required in legacy tiers.	DNA-based PRS, OCT-based retinal or choroidal metrics, and selected dynamic circadian or oxidative biomarkers collected with documented clock time.	Mechanistic bridge and heterogeneity tier for nested substudies; clock-time standardization applies to dynamic biomarkers, not DNA-based PRS.

Abbreviations: AL, axial length; CCT, correlated color temperature; OCT, optical coherence tomography; PRS, polygenic risk score; SE, spherical equivalent.

Supplementary Table S3. Candidate bridge pathways and exploratory mechanistic evidence across exposure domains and ocular tissues

Maps candidate pathways and biomarker classes as exploratory mechanistic evidence for hypothesis generation, not as validation of the proposed framework or clinically deployable biomarkers. Narrative role and replication status reflect the critical narrative synthesis in the manuscript. They guide prioritization for future studies and do not rank biological certainty, imply a validated causal sequence, or validate the proposed framework.

Pathway or biomarker class	Linked exposure domain or context	Tissue, biofluid, or modality	Representative readouts	Narrative role and replication status	Clock-time sensitivity	Representative references
Retinal light-responsive signaling	Outdoor light exposure; timing of light history; evening display light.	Retina; light-responsive pathways; experimental photobiology.	Light-induced signaling pathways, melanopsin-linked responses, dopamine-related candidate pathways, visual cycle signals.	Strong experimental rationale; human support is indirect and mechanistic rather than causal.	High	[26,44,52,53,77]
Circadian endocrine timing signals	Sleep/circadian timing and evening device exposure.	Saliva, blood, or other circadian biofluids.	Melatonin, cortisol, dim-light melatonin onset proxies, internal circadian time markers.	Human experimental evidence is strong for timing effects of displays; direct links to myopia remain indirect.	Very high	[20,21,28,39,69]
Choroidal dynamics and short-term ocular response	Outdoor light history; sleep/circadian timing; near work or defocus sequence.	OCT-based choroidal imaging and short-term physiologic testing.	Subfoveal choroidal thickness, diurnal amplitude, short-term defocus-related change.	Moderate to strong human imaging evidence; replication is improving, but exposure specificity remains incomplete.	High	[58,65,78,85,86]
Scleral ECM remodeling	Shared downstream pathway across outdoor light, near work, sleep/circadian timing, and axial elongation.	Scleral tissue, experimental models, and candidate circulating markers.	Collagen-related transcripts, proteomic ECM signatures, matrix remodeling pathways.	Strong experimental and omics rationale; direct human tissue evidence is limited.	Low to moderate or uncertain	[24,33,53,59,60]

Pathway or biomarker class	Linked exposure domain or context	Tissue, biofluid, or modality	Representative readouts	Narrative role and replication status	Clock-time sensitivity	Representative references
Vascular regulation and choroidal perfusion-related pathways	Daylight history, ocular growth state, and shared downstream response.	Choroid and retina; imaging and selected vascular biomarker studies.	Choroidal thickness dynamics, vasculature-related pathway signals, perfusion-relevant candidate markers.	Emerging human and omics evidence; replication remains limited.	Moderate to high	[58,59,62,65]
Oxidative stress and redox pathways	Sustained near work, circadian disruption, and growth-related stress responses.	Serum, tear, or ocular tissue biomarkers; integrated omics panels.	ROS-related markers, antioxidant pathways, redox-associated omics signals.	Exploratory human biomarker evidence with partial omics support; replication remains limited.	Variable	[59,61]
Integrated omics signatures	Convergence across exposure domains rather than a single exposure-specific route.	Sclera, choroid, retina, blood, or other accessible biofluids.	Transcriptomic, proteomic, metabolomic, and miRNA profiles aligned with ocular growth state.	Exploratory and hypothesis-generating; should be used as a mechanistic bridge and replicated prospectively.	Moderate to high depending on analyte	[59-62]
Genetic liability and pathway-based susceptibility	Upstream susceptibility that may modify associations across exposure domains.	DNA from blood or saliva; pathway-based genetic scores.	PRS, gene set enrichment, and gene-environment interaction signals.	Strong genomic evidence for susceptibility; interaction evidence is growing but less mature than main-effect evidence. Distinct from education-related Mendelian randomization, which addresses upstream causal structure rather than biomarker susceptibility.	Low	[26,27,46-51]

Abbreviations: ECM, extracellular matrix; PRS, polygenic risk score.

Supplementary Table S4. Mapping of operational definitions and future-study hypotheses to measures, study designs, and endpoints

Links each operational construct and priority future-study hypothesis to exposure variables, biospecimens, imaging, genetic context, analytic approach, and falsifiable endpoints for prospective testing.

Operational construct or testable hypothesis	Minimum shared measures	Optional enrichment	Preferred design and analysis	Primary endpoint(s)	Falsifiable result if unsupported
Substitution within a fixed day	Daily time outdoors, near work duration, sleep timing, and device use by time window.	Wearable illuminance sensing and time-stamped device logs.	Prospective cohort or legacy-cohort re-analysis using compositional or time reallocation models.	AL change; cycloplegic SE; incident myopia.	Screen-related time does not alter estimated risk when reallocated across outdoor, sleep, or non-screen near work domains.
Timing and bout structure versus duration alone	Total daily device time plus evening concentration and longest continuous bout.	Ambient light context and viewing distance monitoring.	Repeated measures cohort comparing duration-only models with timing and bout structure models.	AL growth velocity; myopia progression.	Timing metrics do not improve fit, prediction, or interpretability beyond total daily duration.
Outdoor exposure may buffer intensive near work	Daytime outdoor exposure and evening or school-day near-work intensity.	Objective lux data and device or reading timestamps.	Co-exposure categories or prespecified interaction models in cohort data or school trials.	Incident myopia; AL elongation.	Outdoor exposure shows no attenuation of the near work association on additive or multiplicative scales.
Sleep/circadian timing may modify near-work-related risk	Near-work intensity, sleep duration, sleep timing, and timing regularity.	Actigraphy and circadian biomarkers with documented clock time.	Prospective cohort or nested substudy testing interaction between near work and sleep or circadian metrics.	AL change; cycloplegic SE.	Near work associations are unchanged across sleep or circadian strata.

Operational construct or testable hypothesis	Minimum shared measures	Optional enrichment	Preferred design and analysis	Primary endpoint(s)	Falsifiable result if unsupported
Clustered exposure profiles may add information beyond single-domain metrics	Harmonized daylight, near work, and sleep variables sufficient to derive daily or person-level profiles.	Multimodal clustering using sensor, diary, and device data.	Latent class, clustering, or co-exposure modeling with external validation; exploratory until replicated.	Risk discrimination for incident myopia, AL change, or progression.	Clustered exposure profiles fail to improve prediction or do not replicate across cohorts.
Genetic liability may modify exposure effects	Core co-exposure measures plus family history.	PRS and pathway-specific genetic scores.	Cohort-based effect-modification analysis with prespecified genetic strata.	AL growth velocity; incident myopia; high-myopia risk.	Exposure-related associations do not differ across genetic susceptibility strata.
Exploratory mechanistic evidence may align with axial growth velocity	Repeated AL and cycloplegic SE measurements.	Biospecimens, OCT-based choroidal or retinal imaging, and targeted or untargeted omics panels.	Nested mechanistic substudy within cohorts or interventions, prioritized for repeated outcome measurement.	AL growth velocity rather than cross-sectional SE alone.	Biomarker or omics signals do not track repeated AL change or fail replication across cohorts.
Co-exposure	Two or more exposures that occur or are analyzed together.				
Sampling timing may reduce dynamic biomarker heterogeneity	Documented clock time and recent light, near work, and sleep history at sampling for dynamic biomarkers or omics readouts.	Internal circadian time markers or standardized morning sampling.	Prospective biomarker or omics study comparing standardized versus non-standardized sampling context.	Assay variability, reproducibility, and biomarker effect estimates.	Clock-time standardization does not reduce unexplained variance in dynamic biomarkers or improve reproducibility.
School-level candidate intervention package	Protected outdoor time, structured near-work breaks, and reduced evening device concentration.	Objective adherence monitoring, OCT, PRS stratification, or selected biomarkers.	Cluster trial or stepped-wedge design comparing package versus usual practice or outdoor-only strategies.	Incident myopia; AL change; adherence and implementation metrics.	The package is not superior to simpler strategies or produces no differential change in the targeted daily exposure pattern.

Abbreviations: AL, axial length; OCT, optical coherence tomography; PRS, polygenic risk score; SE, spherical equivalent.

Supplementary Table S5. Extended representative human-study inventory across exposure domains and clustered exposure patterns

Complements the main-text representative evidence table with an extended but illustrative inventory of human studies. It is not exhaustive and does not constitute a systematic-review evidence table; evidence-role descriptions are interpretive narrative categories rather than formal grades.

Domain	Representative study	Reference	Narrative evidence role	Design and exposure construct	Outcome(s)	Principal finding and interpretive relevance
Primary domain 1. Outdoor light exposure						
Outdoor school time intervention	He et al., 2015	[11]	Direct interventional or longitudinal human evidence	Randomized clinical trial of additional outdoor time at school.	Incident myopia; myopic shift.	Increasing school-based outdoor exposure reduced incident myopia, supporting a causal preventive role for daylight exposure.
Outdoor recess intervention	Wu et al., 2013	[12]	Direct interventional or longitudinal human evidence	School-based outdoor activity during class recess.	Myopia onset and progression.	Outdoor recess reduced new myopia and supported the practical importance of school timetable design.
Pooled outdoor evidence	Xiong et al., 2017; Kido et al., 2024; Mei et al., 2024; Li et al., 2024	[14]; [15]; [37]; [38]	Direct interventional or longitudinal human evidence	Systematic reviews and meta-analyses of time outdoors and outdoor interventions.	Incident myopia; progression.	The preventive signal is strongest for incident myopia, whereas evidence for slowing progression after onset is smaller and less consistent.
Objective light exposure	Chen et al., 2024	[10]	Objective or prospective supporting evidence	Objective outdoor exposure measurement using smartwatch-based monitoring.	Myopia-related outcomes in children.	Objective light and outdoor metrics strengthen exposure validity and support moving beyond questionnaire-only summaries.
Primary domain 2. Near-work intensity and educational context						
Near work meta-analysis	Huang et al., 2015; Dutheil et al., 2023	[16]; [39]	Cross-sectional or lower-specificity supporting evidence	Systematic reviews and meta-analyses of near work activities.	Myopia or refractive outcomes.	Greater near work is associated with higher myopia risk, but exposure heterogeneity limits interpretation when duration is used alone.
Objective near work and outdoor measurement	Wen et al., 2020	[8]	Objective or prospective supporting evidence	Children studied with objective near work and outdoor exposure measures.	Myopia status and related outcomes.	Viewing distance and directly measured exposure patterns are more informative than crude duration-based summaries.

Co-occurring behaviors	Lanca et al., 2022	[7]	Cross-sectional or lower-specificity supporting evidence	Consortium-based analysis of near work, screen time, and outdoor time in schoolchildren.	Myopia in schoolchildren.	Screen time behaves as a partial marker within a broader behavioral ecology and should be interpreted together with outdoor and near-work measures.
Digital screen dose-response	Ha et al., 2025	[9]	Cross-sectional or lower-specificity supporting evidence	Systematic review and dose-response meta-analysis of digital screen exposure.	Myopia and myopic shift.	Screen duration shows an overall dose-related association with myopia, but duration alone does not identify the biologically relevant feature of exposure.
Education as upstream driver	Cuellar-Partida et al., 2016	[23]	Objective or prospective supporting evidence	Mendelian randomization study of educational attainment and refractive outcomes.	Refractive error or myopia.	Educational attainment shows causal relevance, but the mediating components of education remain unresolved and extend beyond screen use alone.
Education-related displacement	Clark et al., 2023	[43]	Cross-sectional or lower-specificity supporting evidence	Observational analysis evaluating whether outdoor time accounts for part of the education effect.	Myopia-related outcomes.	Time outdoors partly accounts for the education-related signal, supporting mediation or displacement pathways rather than a single exposure explanation.
Lower-certainty modifier. Sleep/circadian timing						
Sleep and myopia review	Liu et al., 2023; Jin et al., 2024; Zhao et al., 2024	[17]; [18]; [19]	Cross-sectional or lower-specificity supporting evidence	Systematic reviews and meta-analyses of sleep duration, timing, and regularity.	Myopia in children and adolescents.	Shorter sleep, later timing, and irregular schedules show modest associations with myopia, but confounding remains an important limitation.
Evening display timing	Chang et al., 2015; Cajochen et al., 2011	[20]; [21]	Translational compatibility evidence	Experimental evening exposure to light-emitting displays or computer screens.	Melatonin, circadian timing, and next-morning alertness.	Evening screen exposure delays circadian timing and suppresses melatonin in adult experiments, supporting the biological distinctiveness of late-day device use while pediatric causal interpretation remains provisional.
Higher-order exposure structure						
Time reallocation and substitution	Lanca et al., 2022; Clark et al., 2023	[7]; [43]	Cross-sectional or lower-specificity supporting evidence	Combined-exposure analyses suggesting that education and screen-related routines should be interpreted within a fixed daily time	Myopia-related outcomes.	Screen or education-related exposure should be interpreted within a fixed daily time budget and not assumed to represent a single causal pathway.

				budget.		
Timing and day structure	Guan et al., 2019	[56]	Cross-sectional or lower-specificity supporting evidence	Study of near-work type and time spent outdoors at different times of day.	Visual acuity and refractive error.	The timing of outdoor and near-work exposure carries additional information beyond total daily duration, supporting a 24-hour architecture perspective.
Outdoor buffering or interaction	Gopalakrishnan et al., 2023; Li et al., 2025	[44]; [45]	Cross-sectional or lower-specificity supporting evidence	Combined or interaction-oriented analyses of outdoor time with reading or near work.	Myopia onset or refractive outcomes.	Outdoor exposure may attenuate the association of intensive reading or near work with myopia, but interaction testing remains limited in most cohorts.
Clustered exposure profiles and combined-exposure modeling	Lanca et al., 2022; Guan et al., 2019	[7]; [56]	Cross-sectional or lower-specificity supporting evidence	Studies showing clustered daily routines rather than isolated exposures.	Refractive outcomes.	Light exposure, near viewing, and daily routines cluster in real life, supporting clustered-profile or co-exposure approaches for descriptive classification or prediction rather than causal inference.
Natural experiments and pandemic-related digitalization						
Home confinement studies	Wang et al., 2021; Laan et al., 2024	[89]; [90]	Objective or prospective supporting evidence	Longitudinal and meta-analytic evidence from COVID-19-related home confinement.	Myopic shift or myopia progression.	Pandemic-related digitalization intensified low outdoor exposure, high near work, and schedule disruption simultaneously, supporting convergence in co-occurring exposure shifts rather than direct evidence of screen-specific causation or causal coupling.

Abbreviations: AL, axial length; SE, spherical equivalent.

Supplementary Table S6. Plain-language glossary of measurement and analytic terms

Defines the specialist measurement and analytic terms used in the manuscript in plain, everyday language.

Term	Plain-language definition
Contextual measurement marker	A variable that helps identify when, where, or how other exposures occur, without being treated as the direct causal exposure.
Daily exposure pattern	The timing, duration, continuity, and sequence of light exposure, near work, and sleep within a day.
Substitution	Replacement of one activity by another within a fixed 24-hour day.
Mediation	A pathway in which one exposure partly transmits the effect of another.
Interaction / effect modification	A situation in which the association of one exposure differs across levels of another.
Clustered exposure profile	A recurring combination of behaviors, such as low outdoor time, long near-work bouts, and delayed sleep timing.
Lower-certainty modifier	A factor with plausible relevance but insufficient evidence to be treated as a primary causal exposure.
Exploratory mechanistic evidence	Biological evidence that can generate hypotheses but does not validate a clinical causal model.

Supplementary Figures

Supplementary Figure S1. Mechanistic compatibility bridge.

The figure links outdoor light, near work, sleep/circadian timing, and digital-device context to candidate ocular-growth pathways and axial elongation. Mechanistic and omics findings are exploratory and hypothesis-generating; they do not validate the framework.

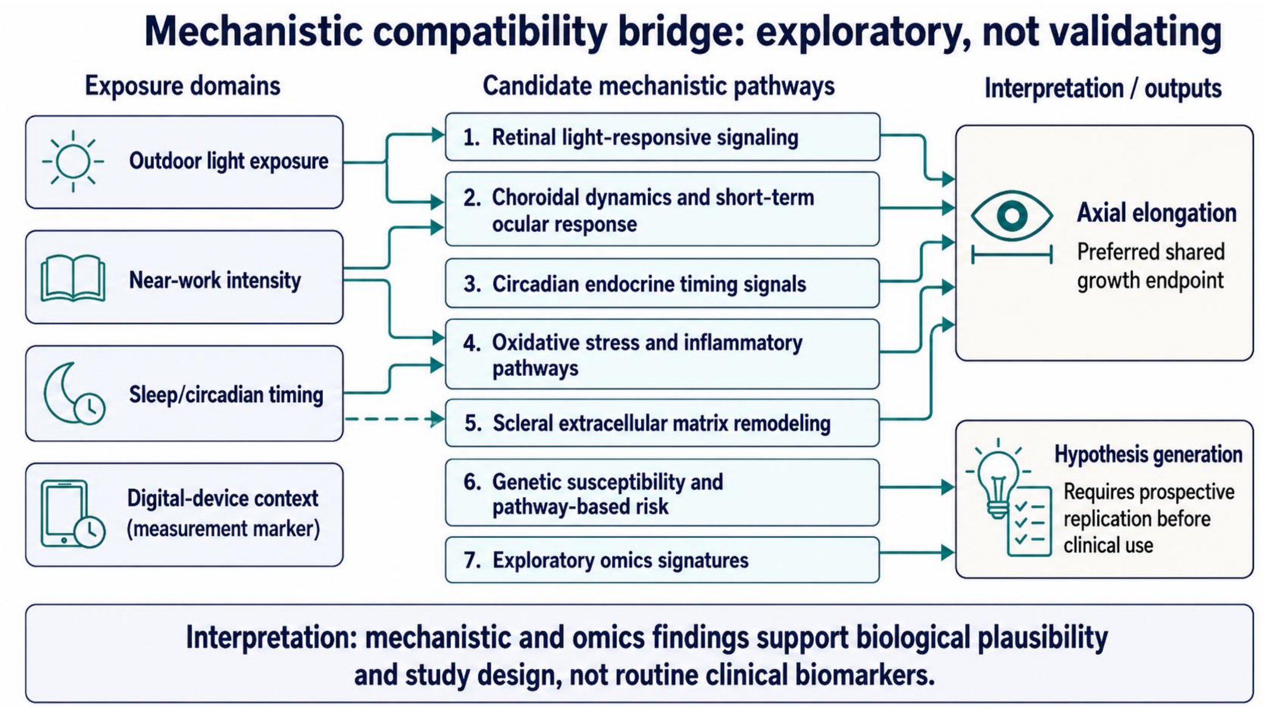

Abbreviations: ECM, extracellular matrix; PRS, polygenic risk score. This figure was generated using an AI tool.

Supplementary Figure S2. Measurement tiers and future-study priorities.

The figure shows legacy-feasible, prospective-standard, and optional-enrichment tiers for testing substitution, mediation, effect modification, and clustered exposure profiles. It does not present a validated causal model.

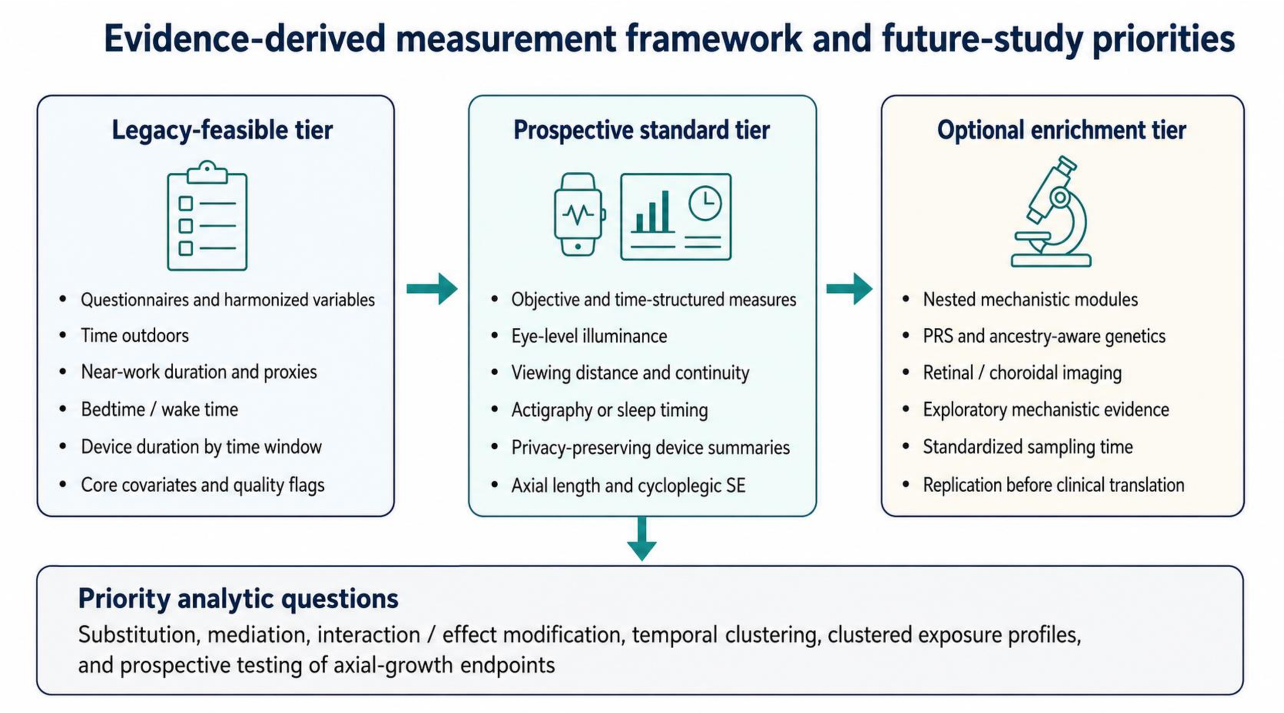

Abbreviations: AL, axial length; PRS, polygenic risk score; SE, spherical equivalent. This figure was generated using an AI tool.
